# Electrospun PCL/gelatin/arbutin nanofiber membranes as potent reactive oxygen species scavengers to accelerate cutaneous wound healing

**DOI:** 10.1093/rb/rbad114

**Published:** 2024-01-12

**Authors:** Mindong Du, Shuhan Liu, Nihan Lan, Ruiming Liang, Shengde Liang, Maoqiang Lan, Disen Feng, Li Zheng, Qingjun Wei, Ke Ma

**Affiliations:** Department of Orthopaedics Trauma and Hand Surgery, The First Affiliated Hospital of Guangxi Medical University, Guangxi Medical University, Nanning 530021, China; Guangxi Engineering Center in Biomedical Materials for Tissue and Organ Regeneration, The First Affiliated Hospital of Guangxi Medical University, Guangxi Medical University, Nanning 530021, China; Guangxi Collaborative Innovation Center for Biomedicine, The First Affiliated Hospital of Guangxi Medical University, Guangxi Medical University, Nanning 530021, China; Guangxi Engineering Center in Biomedical Materials for Tissue and Organ Regeneration, The First Affiliated Hospital of Guangxi Medical University, Guangxi Medical University, Nanning 530021, China; Guangxi Collaborative Innovation Center for Biomedicine, The First Affiliated Hospital of Guangxi Medical University, Guangxi Medical University, Nanning 530021, China; Guangxi Engineering Center in Biomedical Materials for Tissue and Organ Regeneration, The First Affiliated Hospital of Guangxi Medical University, Guangxi Medical University, Nanning 530021, China; Guangxi Collaborative Innovation Center for Biomedicine, The First Affiliated Hospital of Guangxi Medical University, Guangxi Medical University, Nanning 530021, China; Guangxi Engineering Center in Biomedical Materials for Tissue and Organ Regeneration, The First Affiliated Hospital of Guangxi Medical University, Guangxi Medical University, Nanning 530021, China; Guangxi Collaborative Innovation Center for Biomedicine, The First Affiliated Hospital of Guangxi Medical University, Guangxi Medical University, Nanning 530021, China; Department of Plastic & Cosmetic Surgery, The First Affiliated Hospital of Guangxi Medical University, Guangxi Medical University, Nanning 530021, China; Department of Plastic & Cosmetic Surgery, The First Affiliated Hospital of Guangxi Medical University, Guangxi Medical University, Nanning 530021, China; Department of Plastic & Cosmetic Surgery, The First Affiliated Hospital of Guangxi Medical University, Guangxi Medical University, Nanning 530021, China; Guangxi Engineering Center in Biomedical Materials for Tissue and Organ Regeneration, The First Affiliated Hospital of Guangxi Medical University, Guangxi Medical University, Nanning 530021, China; Guangxi Collaborative Innovation Center for Biomedicine, The First Affiliated Hospital of Guangxi Medical University, Guangxi Medical University, Nanning 530021, China; Pharmaceutical College, Guangxi Medical University, Nanning 530021, China; Department of Orthopaedics Trauma and Hand Surgery, The First Affiliated Hospital of Guangxi Medical University, Guangxi Medical University, Nanning 530021, China; Guangxi Engineering Center in Biomedical Materials for Tissue and Organ Regeneration, The First Affiliated Hospital of Guangxi Medical University, Guangxi Medical University, Nanning 530021, China; Guangxi Collaborative Innovation Center for Biomedicine, The First Affiliated Hospital of Guangxi Medical University, Guangxi Medical University, Nanning 530021, China; Guangxi Engineering Center in Biomedical Materials for Tissue and Organ Regeneration, The First Affiliated Hospital of Guangxi Medical University, Guangxi Medical University, Nanning 530021, China; Guangxi Collaborative Innovation Center for Biomedicine, The First Affiliated Hospital of Guangxi Medical University, Guangxi Medical University, Nanning 530021, China; Department of Plastic & Cosmetic Surgery, The First Affiliated Hospital of Guangxi Medical University, Guangxi Medical University, Nanning 530021, China; Pharmaceutical College, Guangxi Medical University, Nanning 530021, China

**Keywords:** arbutin, nanofiber membranes, wound dressing, reactive oxygen species

## Abstract

The presence of excessive reactive oxygen species (ROS) at a skin wound site is an important factor affecting wound healing. ROS scavenging, which regulates the ROS microenvironment, is essential for wound healing. In this study, we used novel electrospun PCL/gelatin/arbutin (PCL/G/A) nanofibrous membranes as wound dressings, with PCL/gelatin (PCL/G) as the backbone, and plant-derived arbutin (hydroquinone-β-d-glucopyranoside, ARB) as an effective antioxidant that scavenges ROS and inhibits bacterial infection in wounds. The loading of ARB increased the mechanical strength of the nanofibres, with a water vapour transmission rate of more than 2500 g/(m^2^ × 24 h), and the water contact angle decreased, indicating that hydrophilicity and air permeability were significantly improved. Drug release and degradation experiments showed that the nanofibre membrane controlled the drug release and exhibited favourable degradability. Haemolysis experiments showed that the PCL/G/A nanofibre membranes were biocompatible, and DPPH and ABTS+ radical scavenging experiments indicated that PCL/G/A could effectively scavenge ROS to reflect the antioxidant activity. In addition, haemostasis experiments showed that PCL/G/A had good haemostatic effects *in vitro* and *in vivo. In vivo* animal wound closure and histological staining experiments demonstrated that PCL/G/A increased collagen deposition and remodelled epithelial tissue regeneration while showing good *in vivo* biocompatibility and non-toxicity. In conclusion, we successfully prepared a multifunctional wound dressing, PCL/G/A, for skin wound healing and investigated its potential role in wound healing, which is beneficial for the clinical translational application of phytomedicines.

## Graphical Abstract

**Figure:**
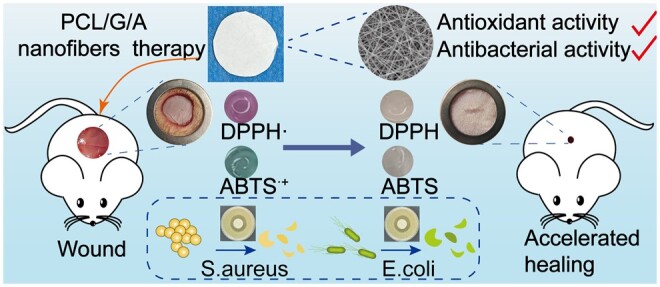

## Introduction

The skin is the first line of defence of the human body and is easily damaged by a variety of external factors, including physical, chemical, biological and mechanical stimuli. Studies have shown that persistent inflammatory responses and excessive production of reactive oxygen species (ROS), including peroxides, superoxides, hydroxyl radicals and singlet oxygen, can prevent wound healing and cause infection [1]. Excessive ROS can alter or degrade extracellular matrix (ECM) proteins, further increasing the levels of inflammatory cytokines and proteases and prolonging the inflammatory response. In addition, excessive ROS in the wound environment has an inhibitory effect on angiogenesis and increases cell death by promoting the excessive production of inflammatory cytokines [2], reactive oxygen intermediates, pro-apoptotic proteins and proteases, leading to a stalled inflammatory phase of healing and impaired wound healing [3, 4]. Therefore, the regulation of ROS levels in the wound microenvironment is critical for skin wound healing.

Antioxidants isolated from plants have shown promising results in wound healing [5, 6]. Arbutin (hydroquinone-β-d-glucopyranoside, ARB) is a hydrophilic active substance extracted from arbutin leaves with antioxidant, antibacterial, anti-inflammatory and hypolipidemic effects, and is widely used in skin care products [7, 8]. Studies have shown that arbutin exhibits a relatively long-lasting ROS-scavenging ability in erythrocytes, reducing oxidative stress by reducing superoxide production and ROS [7]. In prostate cancer, ARB inhibits the inflammatory response and stops prostate progression by regulating the redox environment and reducing ROS levels [9]. The clinical application of ARB is limited by poor stability, ease of removal and poor permeability [10]. Electrostatically spun nanofibrous materials have promising applications in biomedical fields such as wound dressings, skin tissue bioengineering, drug delivery and regenerative medicine because of their high porosity, large specific surface area, high hydrophilicity and ability to mimic the natural fibrous ECM structure in the skin [11]. It has been previously reported that the combination of PCL and gelatin results in a composite scaffold with good cell adhesion and proliferation properties, and good mechanical strength [12]. Electrospun-aligned PCL/gelatin (PCL/G) nanofibres containing ε-polylysine as wound dressings are highly hydrophilic and biocompatible, show high tensile strength and Young’s modulus, broad-spectrum antibacterial properties and promotion of skin wound healing [13]. The electrospinning technique has been shown to be feasible for purposeful mixing with the plant-derived substance EGCG [14]. However, ARB, with a safer history of use as well as an inexpensive price relative to these novel extracts, seems to be a potential option for promoting wound healing. However, ARB, which has a safer history of use, lower expense and wider resource, relative to these novel extracts, seems to be a potentially good substance to combine with spinning for post-wound healing.

In this study, we fabricated PCL/G/A nanofibrous membranes as wound dressings using an electrostatic spinning technique, wherein PCL/G was used as the backbone and plant-derived ARB was used as an effective antioxidant to scavenge ROS and inhibit bacterial infection in wounds. The ability of PCL/G/A nanofibrous membranes to scavenge ROS and the therapeutic effects of PCL/G/A nanofibrous membranes on the repair of rat skin defects were investigated. The incorporation of natural products into electrospun nanofibres to regulate the redox environment may provide novel insights into wound healing.

## Materials and methods

### Fabrication of electrospun PCL/G/a nanofibrous membranes

The PCL/G/A nanofibrous membranes were fabricated via electrospinning. First, PCL and gelatin were mixed at a mass ratio of 7:3 and then dissolved in hexafluoroisopropanol (HFIP, Shanghai Maclean Biochemical Technology Co., Ltd, China). PCL/G mass concentration was 50% (w/v), and the mixture was stirred with a magnetic stirrer at 600 rpm for 12 h at room temperature (26 ± 1°C). Arbutin was dissolved in formic acid (26%, w/v) and stirred at 600 rpm for 1 h at room temperature. Arbutin and PCL/G were mixed at different mass ratios (5:95 and 10:90) and agitated at room temperature for 72 h to obtain electrospun solutions of PCL/G/A at different ratios. For example, 0.35 g of PCL and 0.15 g of gelatin were mixed, 5 ml of hexafluoroisopropanol was added and stirred at room temperature for 24 h, then 100 μl of formic acid mixed with 0.025 g of ARB was added, and a PCL/G/A-5% solution was obtained. The final electrospinning solution was loaded into a 5-ml syringe connected to a stainless-steel 22-gauge blunt needle. The process conditions of electrospinning were as follows: application voltage of 15.0 kV, dispensing rate of 0.7 ml/h and distance between the needle tip and copper roller of 14 cm. After the electrospinning process, the prepared nanofibre film was placed in a vacuum desiccator (LGJ-10C, Foring Technology Development (Beijing) Co., Ltd) for further drying.

### Characterization of composite nanofibrous membranes

The morphologies of the electrospun nanofibrous membranes were observed using scanning electron microscopy (SEM) (TESCAN, VEGA3LMU, Czech Republic) and transmission electron microscopy (HITACHI, H-7650). The diameter distribution of the nanofibers was measured using ImageJ by randomly selecting 50 different fibres. Molecular structure information of electrospun fibre membranes was obtained using Thermo Nicolet FT-IR (Nexus, USA) in the wavelength range of 4000–400 cm^−1^ with a resolution of 4 cm^−1^. The thermal stability of the nanofibrous films was examined using a thermogravimetric analyser at a heating rate of 20°C/min at 800°C. The UV–Vis–NIR spectra were measured using a UV−Vis spectrophotometer (Metash, UV-8000). The mechanical properties of the electrospun fibre membranes were tested using a universal material testing machine (Instron 5943, USA). Wettability studies were performed using the hanging drop method [15], and the static contact angles of the electrospun membranes were measured using a contact angle meter(DSA25, Kruss, Germany) with two parallel measurements at five points for each of the two nanofibre membranes.

The water vapour transmittance was measured according to ASTM Method E96 (ASTM, 1995, American Society for Testing Materials) using the weight reduction method. Three samples were analysed for each experimental group. The formula is as follows:

$$\text{WVTR}\;(g/m^{2}\times 24\;h)=\left(M0\;-\;M1\right)/\left(S\times 24\;h\right),$$

where M0 is the total mass of the experimental material before placing it in the constant temperature and humidity chamber, M1 is the total mass of the experimental material after testing, and S is the area of the bottle mouth.

The samples were cut into discs with a diameter of 12 mm, weighed (W0) and soaked in PBS at 37°C for 24 h. The wet weights (Ww) of the samples were determined. The water absorption formula is as follows [16–18].

Water absorption (%)=(Ww − W0)/W0×100%

### Degradation and drug release of nanofibrous membranes

To study the degradation of nanofibre membranes, scaffolds were immersed in sterile distilled water and incubated at 37°C for various durations (1, 2, 3 and 4 weeks). The nanofibre membrane was dried under a vacuum for 24 h, weighed and photographed under an electron microscope to observe degradation.

Accurately weigh 0.1 g of ARB, and add it to 100 ml of PBS buffer to prepare a 1000 mg/l solution. Dilute it to different concentrations, respectively. According to the literature, 282 nm was finally determined as the maximum absorption wavelength, and the UV absorbance in different concentration solutions was measured, respectively, and the standard curve of ARB released in PBS buffer with pH 7.4 was drawn. ARB-loaded PCL/G nanofiber electrospinning membrane (50 mg) was placed in buffer solution (30 ml) and incubated at 37°C on a shaker and supplemented with fresh buffer. The absorbance at 282 nm was measured by spectrophotometry (model), combined with the ARB standard curve equation, the drug release amount at each time point was calculated, and the release rate could be calculated.

### *In vitro* biocompatibility test

The cytotoxicity of nanofiber membrane to cells was studied by a CCK8 proliferation assay using extraction culture and a live/dead staining method using contact culture. In addition, cell growth on the nanofibre membrane was observed using SEM (TESCAN, VEGA3LMU, Czech Republic).

For the haemolytic performance test of the nanofiber membrane, whole blood was collected from adult SD rats, refrigerated immediately after collection, and blood-related tests were completed within 4 h. The haemolysis test method is based on previous reports [19, 20]. Take a certain amount of whole blood containing anticoagulant (0.109 M citrate, and blood volume ratio 1:9), centrifuge the precipitate, and dilute PBS to obtain red blood cell suspension (RBCS). Three samples were collected from each group, and 1 ml RBCS and 4 ml PBS (pH 7.4) were added to each sample. Negative pairs were treated with 1 ml RBCS and 4 ml PBS (pH 7.4), and positive pairs were treated with 1 ml RBCS and 4 ml deionized water. All samples were incubated in a 37°C water bath at a constant temperature. After centrifugation for 15, 30 and 60 min, the supernatant was collected, the absorbance value at 540 nm was measured, and the haemolysis rate was calculated according to the following formula:

HP (%)=(Dt − Dnc)/(Dpc − Dnc)×100,

where Dt is the absorbance of the sample to be measured, Dpc is the absorbance after mixing deionized water and RBCS, and Dnc is the absorbance after mixing PBS and RBCS.

### Antioxidant test

The antioxidant capacities of the nanofibre membranes were measured using a DPPH kit (Beyotime Institute of Biotechnology). DPPH solutions were prepared in absolute ethanol according to the instructions, and then each membrane sample (2 × 2 cm) was immersed in 1 ml of DPPH solution and incubated at 25°C for 1 h under dark conditions using DPPH solution without membrane as a control. Then, the absorbance of the solution was measured at 517 nm. A lower absorbance corresponds to stronger scavenging activity. The DPPH radical scavenging rates of the membranes were calculated as follows:

DPPH scavenging rate (%)=[(A0 − A1)/A0]×100,

where A0 is the absorbance of the DPPH solution without a fibre membrane as the control, and A1 is the absorbance of the DPPH solution with a fibre membrane.

**Scheme 1.**
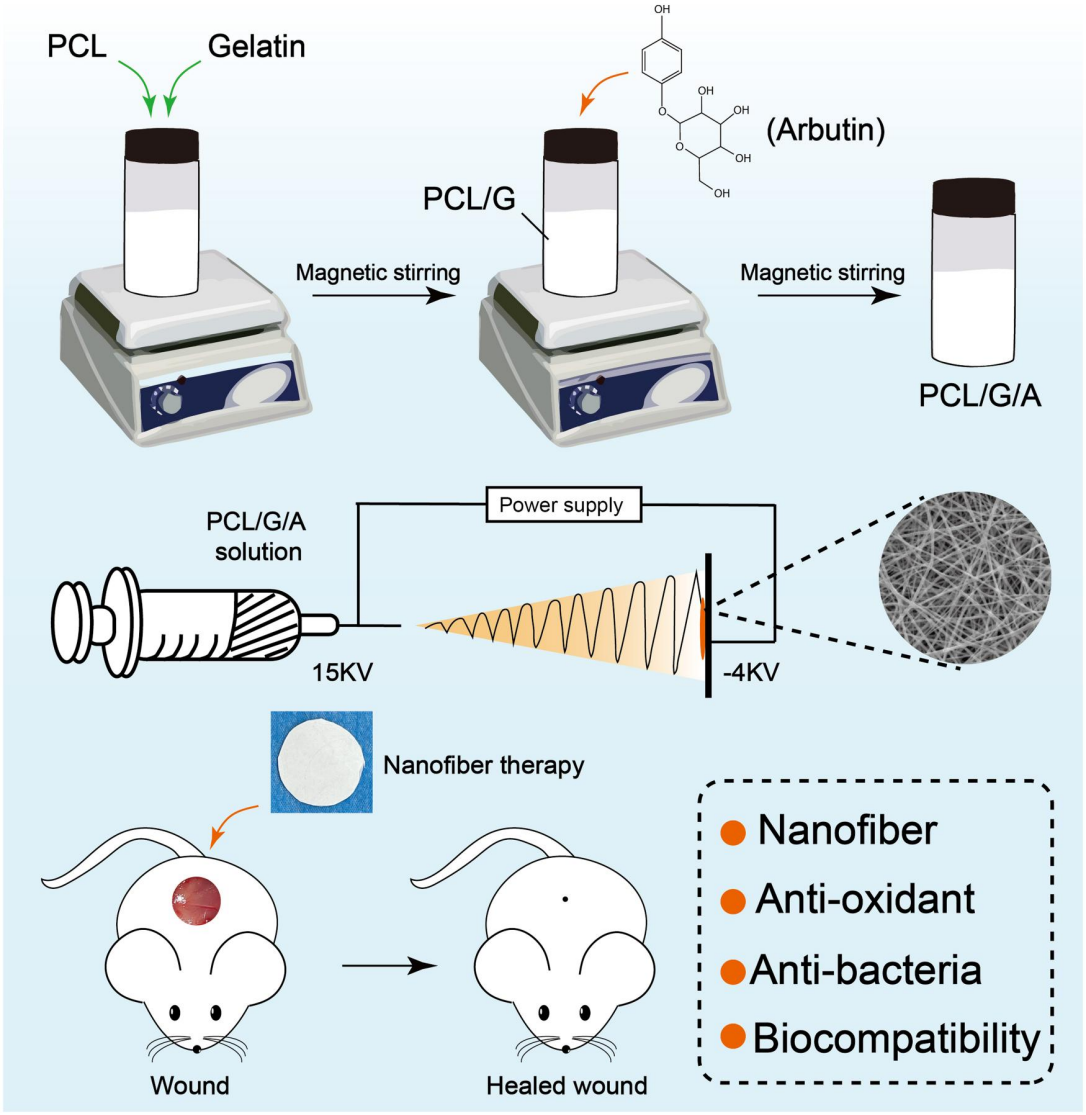
Schematic illustration for the process of preparation of PCL/G/A nanofibrous membranes and mechanism of PCL/G/A nanofibrous membranes promoting skin wound healing in the dorsal skin defect model of rats.

### Antibacterial test

The antibacterial activity of PCL/G, PCL/G/A-5% and PCL/G/A-10% nanofibrous membranes was evaluated using the agar plate diffusion method [21]. Gram-positive *Staphylococcus aureus* (ATCC 43300) and gram-negative *Escherichia coli* (ATCC 25922) were selected as experimental strains. The nanofibre membranes of the PCL/G, PCL/G/A-5% and PCL/G/A-10% groups were cut into discs of 2.7 cm in diameter, sterilized by UV irradiation for 0.5 h and placed on the surface of LB agar plates coated with the strains (1 × 10^6^ CFU/ml). The plates were placed in a 37°C incubator and cultured for 24 h to observe the results. The diameter of the antibacterial ring (including the patch) was measured using Vernier callipers. The concentration of the penicillin in the positive control group was 10 000 U/ml. The experiment was repeated thrice.

### Haemostatic test

Haemostatic tests of the wound dressings were performed using an SD rat liver trauma model. All animal experiments were approved by the Animal Research Ethics Committee of Guangxi Medical University (ethical review approval No. 2023-E253-01). Briefly, after anaesthesia, the rat liver was exposed and stabbed with a puncture needle (18G) to create a model of haemorrhagic liver injury. Blood outflow from the damaged liver was absorbed using a filter paper, and observation time of haemostatic function was uniformly terminated for 5 min. Blood loss was calculated by weighing the filter paper before and after the operation.

### Wound repair

A rat dorsal wound model was established to evaluate the ability of the fibrous dressings to facilitate wound healing. All animal procedures were approved by the Animal Ethics Committee of Guangxi Medical University (ethical review approval No. 2023-E253-01). These guidelines were strictly followed. Female Sprague Dawley (SD) rats (8 weeks, 150–180 g) were anaesthetized by intraperitoneal injection of 3% pentobarbital and then sequentially sterilized on the back with iodophor and 75% alcohol. Subsequently, a round full-thickness skin defect wound (10 mm in diameter) was made on the back. The animals were randomly divided into four groups: saline (control), PCL/G fibre, PCL/G/A-5% fibre and PCL/G/A-10% fibre, and the number of rats in each group was 16. Wound healing was observed at uniform time points (3, 7 and 14 days) after the operation. Digital images were taken with a camera, and the wound area was calculated using ImageJ software. The percentage of wound healing rate was calculated as [A0−Ax/A0] × 100%, where A0 represents the wound area on day 0 and x represents the wound area on days 3, 7 and 14. The result was contributed by mean value of 4 samples in each group. Wound tissues were collected at each time point and sliced, and wound healing was evaluated by haematoxylin–eosin (H&E) staining and Masson immunohistochemical staining. The biocompatibility of the fibres *in vivo* was evaluated by collecting the major organs from each group and creating pathological sections.

### Statistical analysis

The results of quantitative experiments in this study were represented as mean ± standard deviations. Differences between groups were determined using one-way ANOVAs. The value of *P* < 0.05 was considered to be statistically significant (*P < 0.05, **P < 0.01, ***P < 0.001 and ****P < 0.0001).

## Results and discussion

### Synthesis and characterization of PCL/G/a nanofibrous membrane

**Figure 1.**
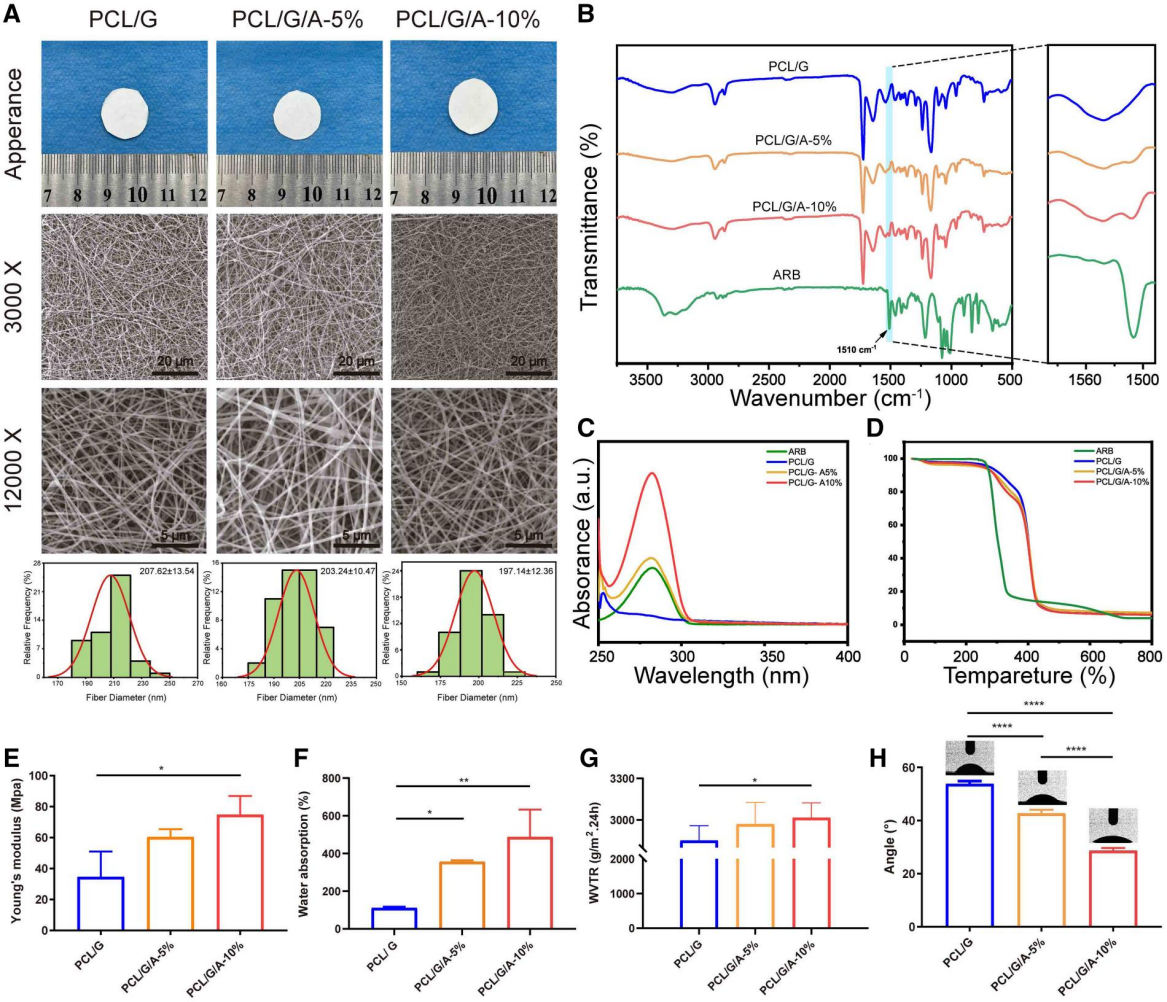
Combined characterization of PCL/G, PCL/G/A-5%, PCL/G/A-10%; (**A**) scanning electron microscope; (**B**) FTIR infrared spectroscopy; (**C**) UV-Visible spectrum; (**D**) thermogravimetric analysis; (**E**) mechanical properties; (**F**) solubility; (**G**) water vapour permeability; (**H**) water contact angle (‘*’ symbol compared between groups, *P < 0.05, **P < 0.01, ***P < 0.001 and ****P < 0.0001).

The morphology of the nanofibre membranes was evaluated by SEM, and the results showed that the synthesized PCL/G nanofibres had a flat and smooth surface, a three-dimensional network structure, and a uniform distribution with mean value diameter of 207.6 ± 13.5 nm (Figure 1A). After loading ARB, the size distribution of the PCL/G/A nanofibres was uniform, no granular aggregates were generated, no phase separation occurred during electrospinning, and the introduction of ARB did not change the structure of the nanofibres. In addition, the diameters of the low-concentration nanofibres were slightly larger than those of the high-concentration nanofibres (Figure 1A). It has been shown that the introduction of active Henna and Gymnema Sylvestree plant extracts into electrospun nanofibres decreases the fibre diameter because of their higher charge density and conductivity [22, 23]. Using FTIR spectroscopy (Figure 1B), the characteristic peak of ARB appeared at 1510 cm^−1^ for the PCL/G/A nanofibre membrane, and this characteristic peak was obvious with the increase in arbutin addition, indicating the successful loading of ARB. The UV–Vis experiments showed the same results. With the addition of ARB, the absorption peak of arbutin appeared at 282 nm and increased with increasing arbutin concentrations (Figure 1C).

Thermogravimetric analysis was used to evaluate the thermal stability of the fibrous membranes. The results showed that PCL/G/A had better thermal stability than ARB alone in the range of 200–500°C (Figure 1D). The good mechanical properties of the nanofibrous membranes contribute to skin wound healing. Previously, Raghavendra *et al*. reported that tensile strength, tensile modulus and toughness were enhanced by adding plant extracts to nanofibers [24]. We examined the mechanical properties of PCL/G, PCL/G/A-5% and PCL/G/A-10% and found that the addition of ARB improved the mechanical properties of PCL/G fibre membranes (Figure 1E). Generally, increasing the fibres’ tensile modulus and strength accompany with decreasing its diameter [25, 26]. According to Lim *et al*. [27], the lamellar packed densely and higher molecular orientation filament structures, in smaller diameter fibres, lead to align tensile forces and improve mechanical properties; increasing fibre diameter, decreasing its structure arrangement, as well as decreasing mechanical properties. Ours studies found that decreasing fibre diameter after loading nanofiber membranes with ARB increasing their mechanical strength, which was consistent with the above studies. So we speculate that plant-derived active substances, by decreasing nanofiber membranes diameter and increasing their structure, thereby improve mechanical properties.

Studies have shown that to maintain a moist wound environment and promote wound healing, an ideal wound dressing should have good air and vapour permeability, as well as absorbency to reduce evaporative water loss from the wound, drug absorption, exudate control and reduce the risk of bacterial infection [28]. To evaluate the water absorption and air/water vapour transport capacities of the electrospun nanomembranes, we examined their swelling, water permeability and water contact properties of the electrospun membranes. The swelling rate of the PCL/G/A nanofibre membranes was higher than that of the pure PCL/G nanofibre membranes, and the swelling rate gradually increased with an increasing ARB ratio (Figure 1F). High swelling rates have been reported to facilitate the sustained release of bioactive substances, and experimental results have shown that ARB can be transported and sustained in PCL/G/A nanofibres [29]. The water vapour transmission rate of the fibre membrane was above 2500 g/(m^2^ × 24 h), which is very close to the water vapour transmission rate required for wound healing (2000–2500 g/(m^2^ × 24 h)), indicating that the PCL/G/A nanofibre membrane has a high water vapour transmission rate (Figure 1G). The water vapour transmission rates of the nanofibre membranes containing ARB were higher than those of the membranes without ARB. The water vapour transmission rate of the PCL/G/A nanofibre membranes increased with the loading of ARB, which may be related to the hydrophilicity of ARB itself. The water vapour transmission rate facilitates gas exchange, such as oxygen and water vapour, during wound healing, which is necessary for cell proliferation, while preventing wound dehydration. The hydrophilic nature of a wound dressing is a key factor in maintaining a moist environment and absorbing exudate is the hydrophilic nature of the wound dressing [30]. Figure 1H shows the variation in the contact angle of nanofibre membranes with different cross-links. As seen in Figure 1H, the water contact angle of PCL/G nanofiber membranes gradually decreased from 53.82–42.68 and 28.66 degrees with the increase of ARB content. The results indicate that the hydrophilicity of the nanofibre membrane surface was significantly enhanced with an increase in the ARB content in the nanofibre membrane (*P* < 0.05). Hydrophilic wound dressings absorb wound exudates in healthy wound-healing environments. PCL is a hydrophobic polymer, and when gelatin is added, the dressing becomes hydrophilic (i.e. the water contact angle decreases). The addition of ARB, a hydrophilic compound with multiple OH groups, reduced the water contact angle and increased the hydrophilicity of the dressing. These results are consistent with those reported by Unalan *et al*. that hydrophilic groups and polar components of plant-derived active substances can enhance the hydrophilicity of PCL/G nanofibers [31]. This suggests that the PCL/G/A nanofiber membrane has good hydrophilic and breathable properties that help accelerate wound healing.

### Degradation and drug release of PCL/G/a nanofiber membrane *in vitro*

**Figure 2.**
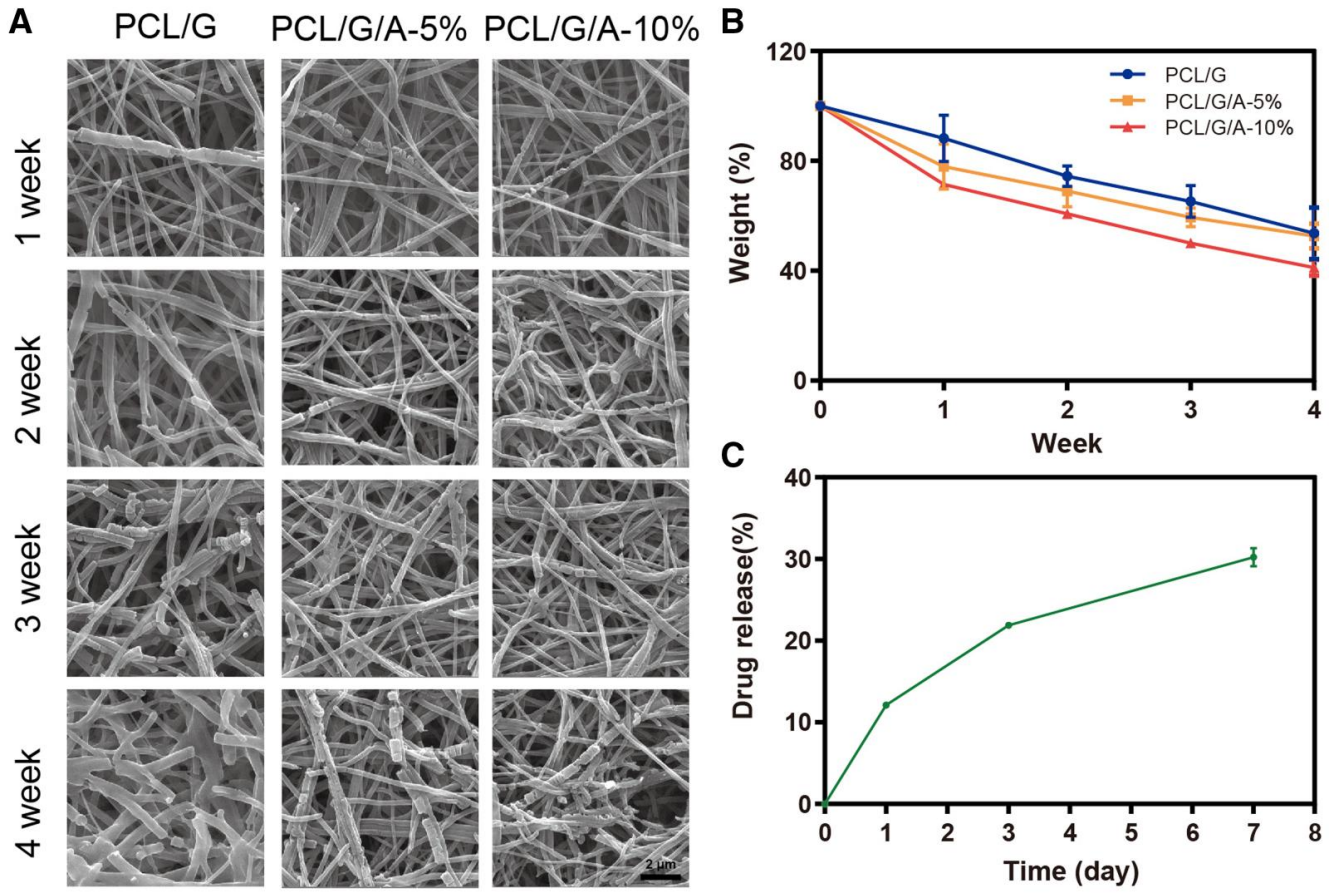
Degradation and drug release of PCL/G, PCL/G/A-5%, PCL/G/A-10%. (**A, B**) degradation of PCL/G, PCL/G/A-5%, PCL/G/A-10% components at different time points under transmission scanning electron microscopy; (**C**) Release of ARB from PCL/G/A-10% in PBS (pH 7.4) at different time points (‘*’ symbol compared between groups, *P < 0.05, **P < 0.01, ***P < 0.001 and ****P < 0.0001).

The *in vitro* degradation experiments of the nanofibre membranes showed that the PCL/G nanofibre membranes had a good ARB degradation rate, and the degradation rate further increased with an increase in ARB content (Figure 2A and B), probably because the addition of ARB increased the hydrophilicity of the fibre membranes and improved the contact between the fibres and water. Within 4 weeks, the fibres degraded to an initial 53.65% when ARB was not loaded. When the ARB content of ARB increased to 5%, the fibre degraded to 52.63%. When the ARB content of ARB increased to 10%, the fibres degraded the fastest, reaching 41.11%. ARB possesses a wide range of advantages in terms of pharmacological properties, including anti-inflammatory, antioxidant and antibacterial properties [32–35]. However, ARB has inherent limitations, such as instability and low bioavailability, which seriously hinder its clinical application [36]. In this study, we evaluated the release behaviour of PCL/G/A. The ARB drug release curves are shown in Figure 2C. At pH 7.4, the fibrous membranes of both ARB concentrations showed a burst of early and rapid release in the first 2 h, followed by a slow and sustained release of ARB for 1–7 days, a result consistent with the results of Karuppannan *et al.* [37]. This may be due to the high specific surface area of the nanofibres, and the ARB drug diffuses rapidly into the medium in the early stage. Later, owing to the special structure of nanofibres, the drug is wrapped by the fibre membrane, causing slow release [38]. Ramalingam *et al*. showed that the active extracts of plant origin showed explosive release in the initial phase and reduced bacterial colonization in wounds [39]. The results of this study show that PCL/G/A has good degradability and can achieve long-term and controlled release of ARB, improving drug stability and bioavailability, which are conducive to sustained drug performance and antibacterial effects at the wound site.

### Cell proliferation, adhesion and haemolysis *in vitro*

**Figure 3.**
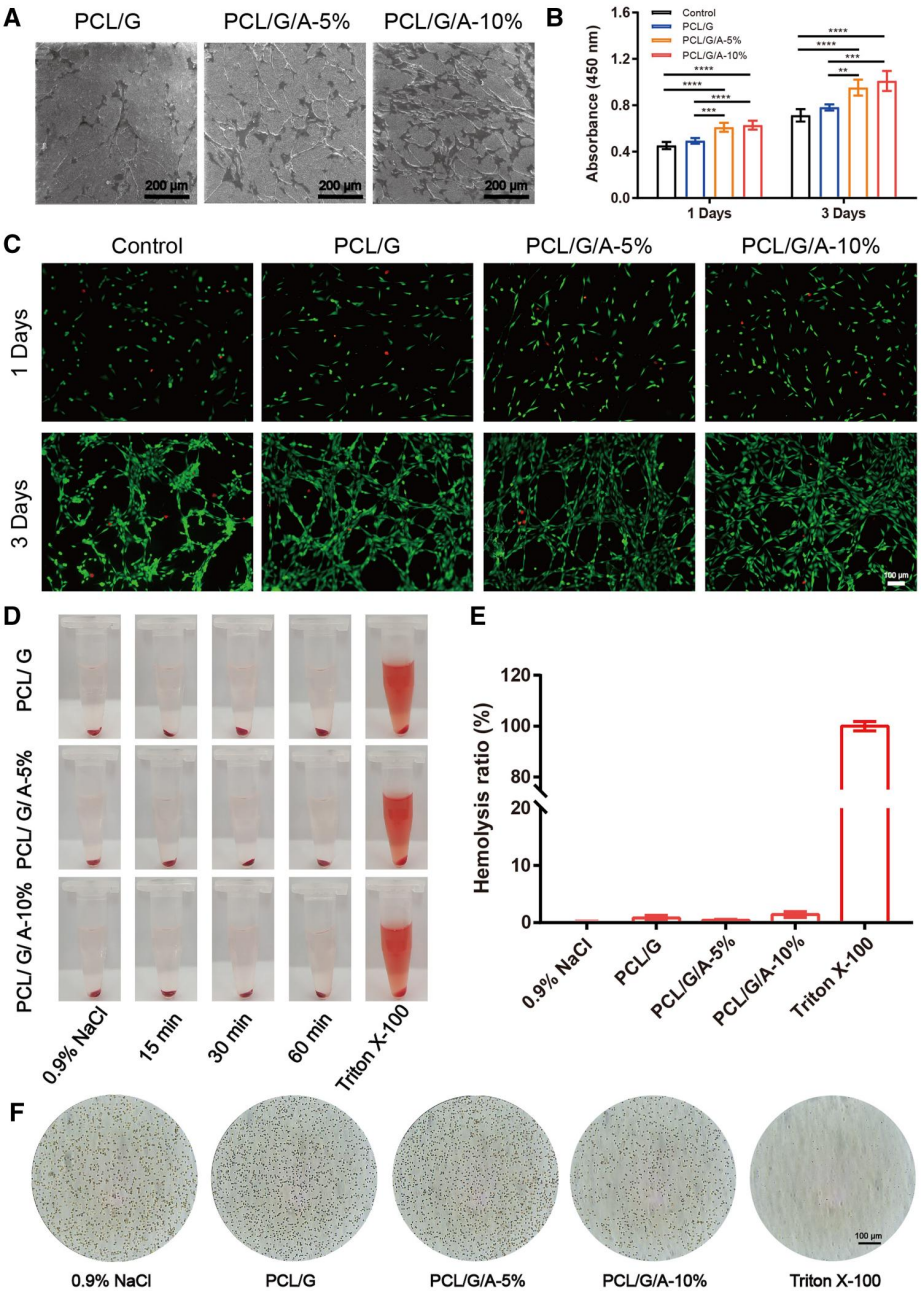
(**A**) Cell adhesion of mouse fibroblast lineage NIH3T3 cells on PCL/G, PCL/G/A-5%, PCL/G/A-10%; (**B**) mouse fibroblasts cultured in each group of nanofibrous membranes after 24 and 72 h of leachate cell proliferation CCK8 assay; (**C**) live-dead immunofluorescence of mouse fibroblasts cultured in various groups of nanofibrous membranes; (**D**) *in vitro* haemocompatibility evaluation of nanofibrous membranes. Gross view of haemolysis in each group; (**E**) statistical comparison of haemolysis in each group; (F) the morphology of erythrocytes was observed under a microscope (‘*’ symbol compared between groups, *P < 0.05, **P < 0.01, ***P < 0.001 and ****P < 0.0001).

Electrospun nanofiber membranes have been reported to provide a good microenvironment for cell proliferation and adhesion and contribute to wound healing because they can mimic the biological structure and function of the ECM [40]. In this study, cell adhesion and proliferation on electrospun fibrous membranes were evaluated using the CCK8 assay. Electron microscopy revealed that the cells on the nanofibrous membranes were polygonal in shape with uniform cell growth. Meanwhile, the expansion ability of adherent cells on PCL/G/A-10% was enhanced, and the number of cells increased. The addition of ARB enhanced cell adhesion (Figure 3A). CCK8 results showed that after mouse fibroblasts were exposed to each set of nanofiber membranes for 24 and 72 h, the proliferation capacity of leachate cells increased with increasing concentrations of ARB (Figure 3B). To further investigate the effect of the nanofibrous membrane on cell proliferation, live and dead cell staining was done. As shown in Figure 3C, the proportion of live cells (green) was much higher than that of dead cells (red) across all cell membranes. ARB has been reported to mobilize fibroblasts at wound sites by activating the insulin/IGF-1 signalling pathway, thereby promoting cell migration and proliferation [41]. The results showed that the nanofibres containing ARB (PCL/G/A-5% and PCL/G/A-10%) had higher cell viability than the nanofibres without ARB (PCL/G) (Figure 3C). The highest cell viability was observed for PCL/G/A-10%, followed by PCL/G/A-5%. Assessing haemolysis *in vitro* is a critical first step in determining the safety of nanofibrous membranes. A haemolysis test was used to preliminarily evaluate the biocompatibility and feasibility of PCL/G/A wound administration. Figure 3D–3F shows that the haemolysis rates of PCL/G, PCL/G/A-5% and PCL/G/A-10% *in vitro* were less than 5%, indicating that nanofibre membrane wound administration has excellent haemocompatibility and safety. It can be concluded from the above results that nanofibre membranes with both ARB concentrations have good biocompatibility and haemocompatibility, while nanofibre membranes with an ARB concentration of 10% seemed to be more favourable for cell attachment and proliferation.

### *In vitro* antioxidant activity

**Figure 4.**
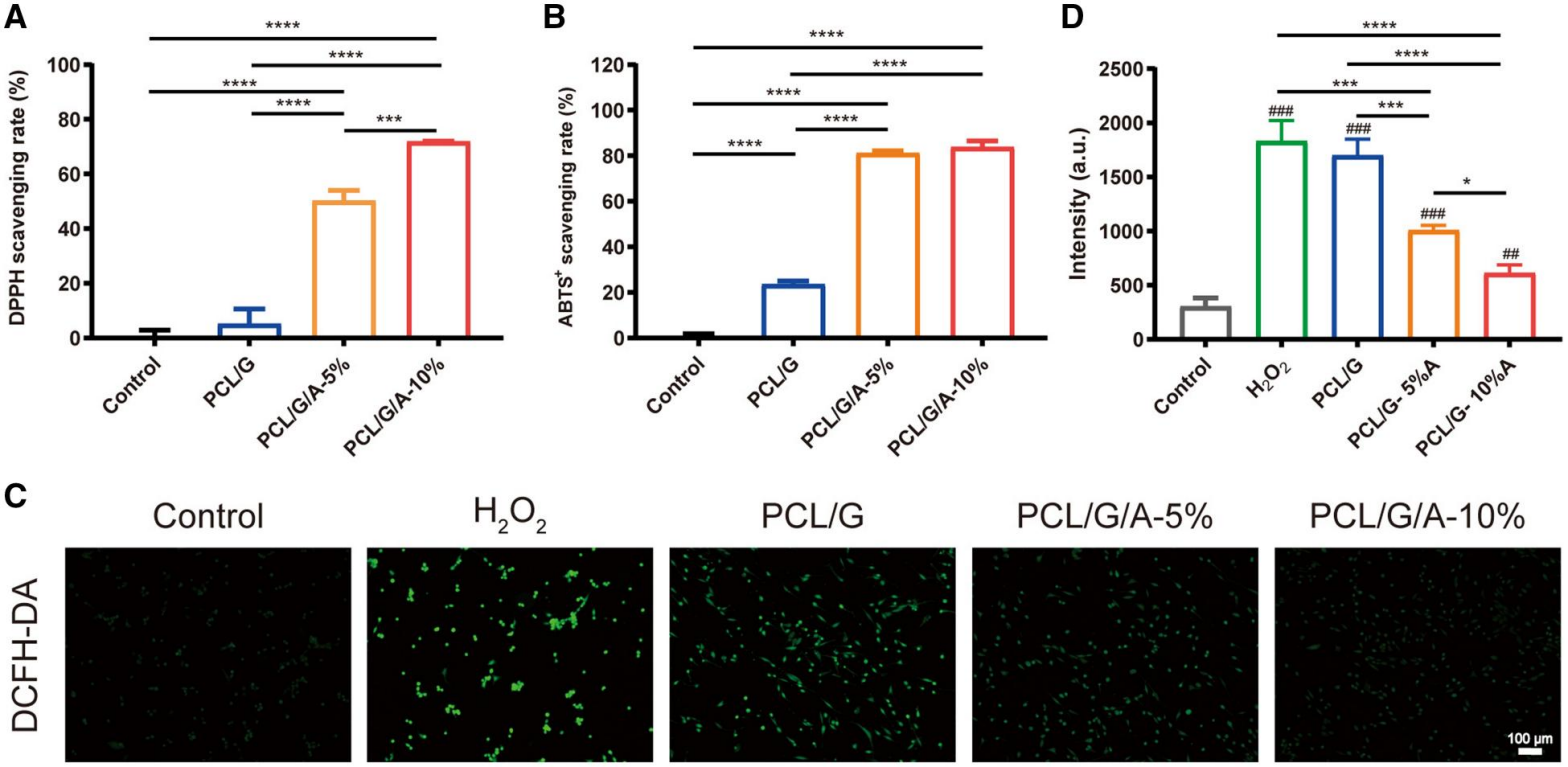
Antioxidant capacity of nanofibers to scavenge ROS: (**A**) DPPH clearance; (**B**) ABTS^+^ clearance; (**C, D**) DCFH-DA fluorescence intensity evaluation and immunofluorescence images (‘*’ symbol compared between groups, *P < 0.05, **P < 0.01, ***P < 0.001 and ****P < 0.0001).

Increased oxidative stress is a major challenge in the wound-healing process. Increased production of ROS in wounds leads to the death and migration of keratinocytes, which, in turn, leads to abnormal epidermal migration and incomplete wound closure [42]. This implies that reducing oxidative stress is essential for promoting wound healing [43]. Because ARB has good antioxidant activity, the antioxidant activity of nanofibrous membranes loaded with ARB was systematically investigated. The DPPH clearances of PCL/G, PCL/G/A-5% and PCL/G/A-10% were 5.24, 50.28 and 71.94%, respectively (Figure 4A), indicating that the DPPH radical scavenging activity of the PCL/G/A nanofibre membranes increased with increasing ARB concentration. The cation scavenging activity of ABTS radicals further confirmed that PCL/G/A had a significantly enhanced free radical scavenging ability compared to the PCL/G fibre membranes (Figure 4B). PCL/G, PCL/G/A-5% and PCL/G/A-10% exhibited 23.67, 81.27 and 83.83% ABTS radical scavenging activities, respectively. Additionally, ROS probes were used to assess the ROS-scavenging effects of the PCL/G/A nanofibrous membranes. The results revealed that the PCL/G/A nanofibre membranes significantly scavenged ROS compared with the pure PCL/G fibre membranes (Figure 4C and D). This result further confirmed the role of ARB in antioxidant activity, cell protection and promotion of cell proliferation, consistent with previous findings [43, 44].

### *In vitro* antibacterial ability

**Figure 5.**
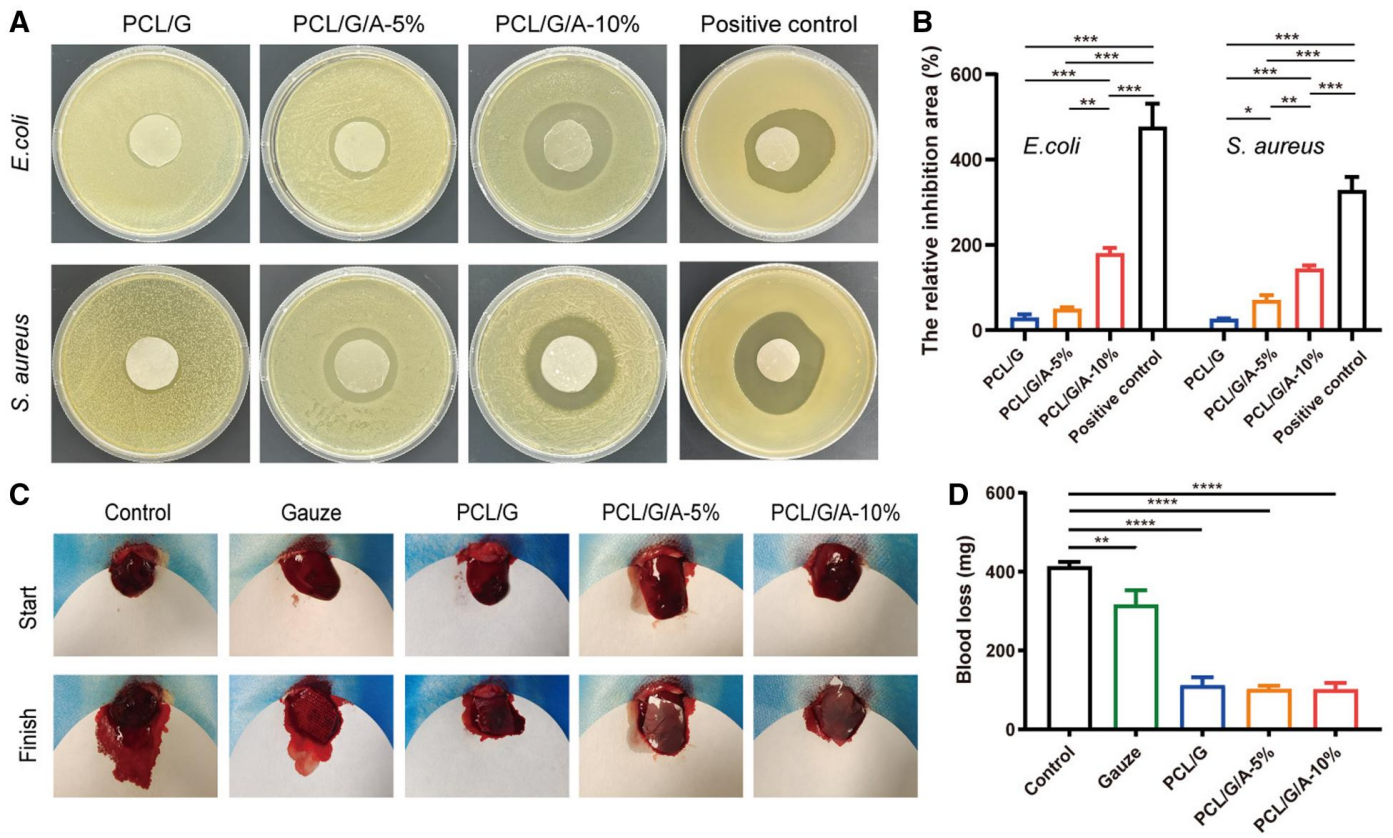
Antibacterial test and haemostasis test. (**A**) Representative images showing the antibacterial ability of different nanofiber membrane on *S. aureus* and *E. coli*. (**B**) Statistical analysis of the antibacterial test’ results. (**C**) Amount of blood loss from a liver wound in different group. (**D**) Statistical analysis was performed by one-way ANOVA (‘*’ symbol compared between groups, *P < 0.05, **P < 0.01, ***P < 0.001 and ****P < 0.0001).

In addition to good biocompatibility, degradability and water permeability, wound dressings should also have antibacterial properties to effectively prevent pathogenic infection of wounds [13, 45]. The most common pathogens that cause skin wound infections are *S. aureus* and *E. coli* [46]. In this study, the antimicrobial performance of the nanofibre membranes was examined using the agar plate diffusion method, and the diameters of the inhibition circles of the PCL/G, PCL/G/A-5% and PCL/G/A-10% nanofibre membranes were measured and analysed (Figure 5A). The results showed that the antibacterial effects of PCL/G, PCL/G/A-5%, PCL/G/A-10% and penicillin were 29.77, 50.33, 180.93 and 477.3% against *S. aureus*, respectively, and 27.40, 70.97, 144.62 and 328.9% against *E. coli*, respectively, with PCL/G/A-5% and PCL/G/A-10% showing the better antibacterial performance than PCL/G against the two experimental strains (Figure 5B). As ARB has an antibacterial pharmacological effect [9], the antibacterial effect may be more pronounced after the concentration is increased. The PCL/G/A nanofibre film inhibited *S. aureus* and *E. coli*, which is useful for the effective suppression of wound infection and can accelerate wound healing.

### Evaluation of haemostatic capacity

Haemostasis marks the beginning of the wound-healing process, and rapid and effective haemostasis can reduce blood loss and prevent inflammation, thereby promoting wound repair. Therefore, we selected specific areas of the liver of SD rats for the incision to establish a liver haemorrhage model and explore the haemostatic ability of the nanofibre membranes *in vivo*. As shown in Figure 5C, the control rats that did not receive nanofibre treatment experienced substantial blood loss, as evidenced by the large blood spots on the filter paper placed over the bleeding site. However, when the nanofibres were applied to the wounds, blood loss ceased rapidly, as evidenced by the very small amount of blood on the filter paper. The haemostatic properties of the gauze were inferior to those of the nanofibres. As shown in Figure 5D, the haemostatic ability of the nanofibres was independent of the addition of ARB. *In vitro* experiments demonstrated that nanofibre membranes have good hydrophilic and haemolytic properties and that the reduction of water in the blood leads to slow blood flow, thus providing haemostasis.

### Wound healing *in vivo*

**Figure 6.**
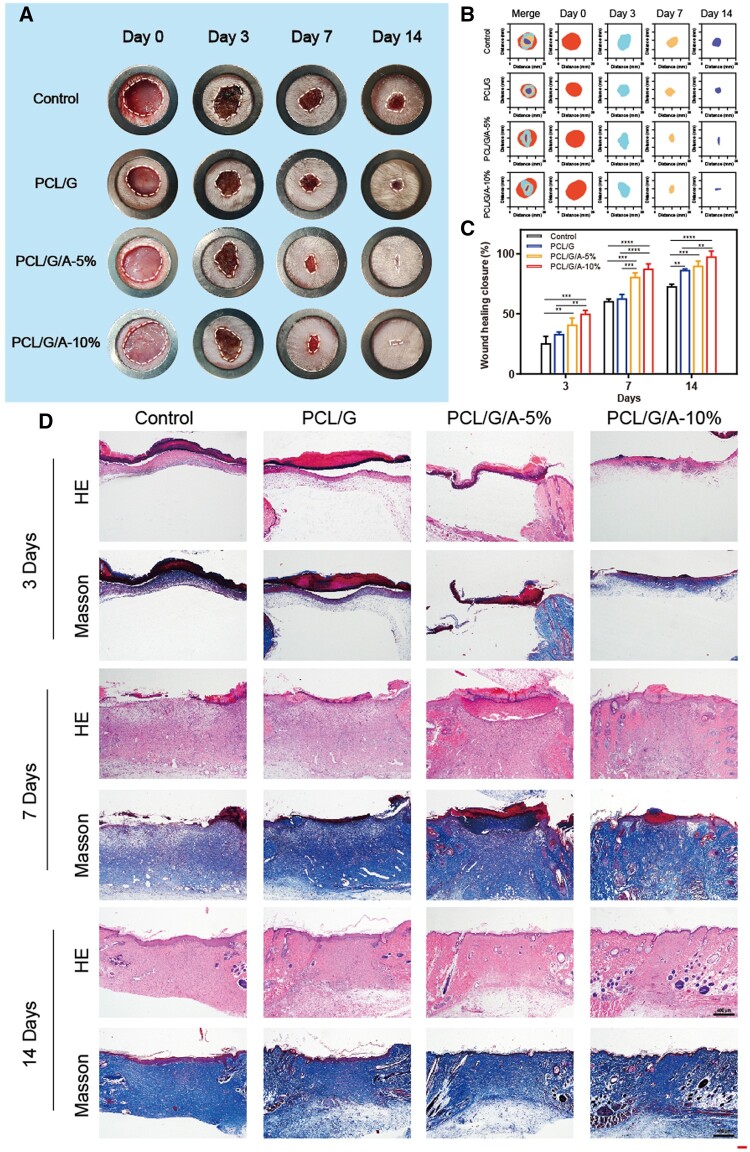
A comprehensive evaluation of wound healing by nanofibrous membranes on days 3, 7 and 14; (**A**) gross observation of wound closure; (**B**) analysis of wound healing marks; (**C**) statistical analysis of wound area; (**D**) histological staining evaluation (‘*’ symbol compared between groups, *P < 0.05, **P < 0.01, ***P < 0.001 and ****P < 0.0001).

**Figure 7.**
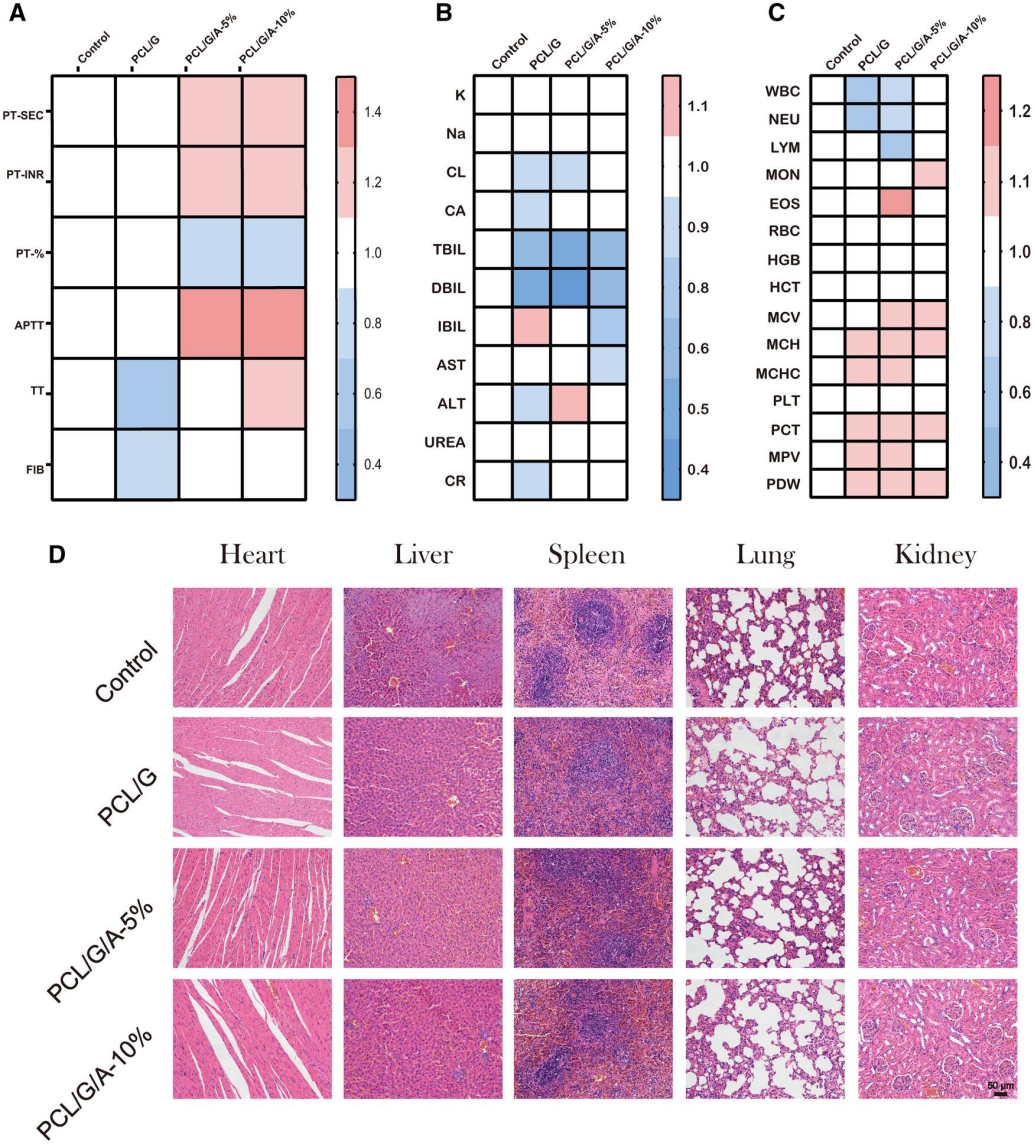
Evaluation of *in vivo* biocompatibility and safety: (**A**) coagulation evaluation; (**B**) electrolyte liver and kidney function evaluation; (**C**) evaluation of routine blood indicators; (**D**) HE staining of the heart, liver, spleen, lungs and kidneys of major organs of animals.

To explore PCL/G/A nanofibrous membrane-mediated wound healing *in vivo*, we established a wound model with full-thickness skin defects to explore *in vivo* PCL/G/A nanofibrous membrane-mediated wound healing. The efficacy and safety of the nanofibre membranes were assessed by observing wound closure (Figure 6A–C), histological staining assay (Figure 6D) and *in vivo* biocompatibility (Figure 7A–D) on days 3, 7 and 14. The results of wound closure after 14 days of treatment with ARB-containing nanofibre membranes (Figure 6A–C) showed that PCL/gelatin/ARB accelerated wound healing compared to the control group, and on day 3, the advantage of adding ARB was not significant. On day 7, the ARB-treated group with added ARB clearly showed better wound healing than the other groups did. In addition, PCL/G/A-10% group healed faster than the other groups, with a wound closure rate of 97.82% by day 14 (*P* < 0.05) (Figure 6C). Wound healing progresses through the stages of scar proliferation and remodelling and can be assessed by the amount of collagen deposited at the wound site, which can be coloured blue by the dye [47]. Histological assessment using Masson staining is shown in Figure 6D. Compared with the control group, after 14 days of PCL/G/A-10% treatment, the wounds showed some basic epithelial structures, abundant collagen fibres and a large number of fibroblasts. Furthermore, collagen deposits in the regenerated tissue were similar to those in the adjacent normal skin tissue. In contrast, the control group lost the normal arrangement of cells and the regenerated tissue was almost scar tissue. The histological results were consistent with those of the closure. Biocompatibility is important for the safe application of nanofibrous membranes *in vivo*. The biocompatibility of the PCL/G/A nanofibre membranes was evaluated using a subcutaneous implantation model in SD rats. The results showed that SD rats treated with PCL/G, PCL/G/A-5% and PCL/G/A-10% nanofibrous membranes exhibited coagulation and routine blood parameters comparable to those of healthy mice 14 days after subcutaneous implantation, with no significant differences (Figure 7A and C). Metabolic screening also showed no significant changes in liver or kidney function in the SD rats (Figure 7B). In addition, H&E staining showed no chronic pathological or physiologically active toxicities in the major internal organs of SD rats treated with the PCL/G, PCL/G/A-5% and PCL/G/A-10% nanofibrous membranes (Figure 7D). These results suggest that ARB-doped nanofibres have good *in vivo* biocompatibility and no potential systemic toxicity and may promote wound healing by reducing ROS species and antioxidant production.

## Conclusion

In summary, we successfully prepared nanofibrous membranes containing plant-derived ARB bioactivities by electrospinning for antibacterial, haemostatic, antioxidant and wound-healing applications. The results of *in vitro* studies showed that the prepared PCL/G/A nanofibrous membranes had a smooth fibrous structure. Compared to the scaffold PCL/G alone, PCL/G/A exhibited good wettability, water vapour transmission rate, swelling, biocompatibility, haemostasis, antibacterial ability and ROS scavenging ability, and the 10% concentration of ARB nanofibrous membrane was superior to the 5% concentration of the ARB nanofibrous membrane. *In vivo,* animal experiments have shown that PCL/G/A nanofibrous membranes may accelerate wound healing by reducing the duration of the inflammatory response through ROS scavenging and antibacterial activity. This study posits that PCL/G/A nanofibrous membrane can promote wound healing and has good histocompatibility, which has a clinical application prospect in the field of wound healing. We also propose the development of multifunctional wound dressings based on electrospun nanofibres to explore the efficacy of ARB in clinical wound repair and their therapeutic potential in soft and hard tissue regeneration.
